# Actin Fusion Proteins Alter the Dynamics of Mechanically Induced Cytoskeleton Rearrangement

**DOI:** 10.1371/journal.pone.0022941

**Published:** 2011-08-05

**Authors:** Martin Deibler, Joachim P. Spatz, Ralf Kemkemer

**Affiliations:** 1 Department of New Materials and Biosystems, Max Planck Institute for Intelligent Systems, Stuttgart, Germany; 2 Department of Biophysical Chemistry, University of Heidelberg, Heidelberg, Germany; Dalhousie University, Canada

## Abstract

Mechanical forces can regulate various functions in living cells. The cytoskeleton is a crucial element for the transduction of forces in cell-internal signals and subsequent biological responses. Accordingly, many studies in cellular biomechanics have been focused on the role of the contractile acto-myosin system in such processes. A widely used method to observe the dynamic actin network in living cells is the transgenic expression of fluorescent proteins fused to actin. However, adverse effects of GFP-actin fusion proteins on cell spreading, migration and cell adhesion strength have been reported. These shortcomings were shown to be partly overcome by fusions of actin binding peptides to fluorescent proteins. Nevertheless, it is not understood whether direct labeling by actin fusion proteins or indirect labeling via these chimaeras alters biomechanical responses of cells and the cytoskeleton to forces. We investigated the dynamic reorganization of actin stress fibers in cells under cyclic mechanical loading by transiently expressing either *egfp-Lifeact* or *eyfp-actin* in rat embryonic fibroblasts and observing them by means of live cell microscopy. Our results demonstrate that mechanically-induced actin stress fiber reorganization exhibits very different kinetics in EYFP-actin cells and EGFP-Lifeact cells, the latter showing a remarkable agreement with the reorganization kinetics of non-transfected cells under the same experimental conditions.

## Introduction

Many cells *in vivo* are exposed to mechanical forces. They are able to sense these external stimuli and convert them to intracellular signals by a complex mechanism termed “mechano-transduction”. In this way mechanical cues can influence or regulate the biological behavior of cells on different time scales [1]. The cytoskeleton is a crucial element in force-induced cell responses [2]. First, it is proposed to be an important component of sensing mechanisms, which translate the mechanical cues into biochemical signals [3], [4]. Second, the cytoskeleton is a stress-bearing structure that maintains cellular integrity and morphology and it serves as an actuator through adaption of its architecture [5], [6]. Due to these important properties, particular emphasis in cellular biomechanics has been given to the acto-myosin system [2], [7]. In order to observe the actin cytoskeleton in living cells the transgenic expression of fluorescent proteins fused to actin is a generally accepted method [8]. Nonetheless, adverse effects of GFP-actin fusion proteins on cell spreading, migration and cell adhesion strength have been reported [9]. Riedl et al. circumvented these particular drawbacks by fusing GFP to a short (17 aa) yeast derived peptide (Lifeact), which proved to efficiently associate with F-actin without altering cellular behavior in migration processes [10]. Lifeact was shown to co-localize with GFP-actin, but comparison of the two phenotypes to phalloidin-stained microfilaments waits to be performed. Despite partly shared signaling pathways and mechanisms, actin reorganization in migration processes and force-induced adaptations are difficult to compare. Differences in acting forces, kinetics and structural changes are obvious. However, a detailed and quantitative analysis of the impact of the labeling method on morphological features and the dynamical behavior of the actin cytoskeleton in force-induced processes is missing.

A common method to investigate responses of cells and the cytoskeleton to extrinsic forces is to use mechanically expandable culture substrates [11]–[13]. By stretching the substrate the mechanical deformation is transferred to the adherent cells. Exposed to uniaxial periodic tensile strain, the cell body and its contractile acto-myosin system tend to align in a perpendicular direction with respect to the stretch axis [13]–[15]. These reorganization processes occur within several minutes and coincide with the reorganization of cellular adhesive contacts [16], [17]. However, most studies investigated such responses only in a state-to-state like manner, not observing the dynamics of them. Due to this shortcoming, there is only little information on the kinetics of acto-myosin adaptions upon mechanical stimulation. Only few recent studies tried to capture the dynamic reorganization of the actin cytoskeleton [13], [18] with GFP-actin fusion proteins.

In this work, we investigated if the labeling of actin, either directly by fluorescent proteins or indirectly via Lifeact, alters the morphology or the force-induced dynamic reorganization of the acto-myosin system. A custom-built stretching device allowing for live cell imaging was used to subject adherent rat embryonic fibroblasts (REF52wt) to periodic tensile strain. We followed the reorganization of the acto-myosin system over time and found significant differences in the reorganization kinetics of the actin stress fiber system depending on the labeling method. As such, our results clearly demonstrate that the choice of the labeling method can drastically alter the kinetics of the actin cytoskeleton behavior in biomechanically motivated cell experiments.

## Results

In order to investigate the dynamic actin cytoskeleton reorganization upon cyclic stretching of cells *egfp-Lifeact* or *eyfp-actin* was transiently expressed in rat embryonic fibroblasts (REF52wt). The cells were cultured on flexible, fibronectin-coated polydimethylsiloxane (PDMS) substrates that were subjected to uniaxial cyclic tensile strain (CTS) with a frequency of 4 s^−1^ and an elongation of 8% [19]. Force-induced changes in cell body and actin stress fiber orientation were observed by live cell phase contrast and fluorescence microscopy. Via these methods we recorded movies of mechanically stimulated cells over 100 min with a temporal resolution of 5 min and 10 min respectively (Movie S1, S2 in Supporting Information). In order to avoid strong impact of the expression level of the two transfected constructs, we selected cells having a similar integrated brightness at matching acquisition times.

**Figure 1:**
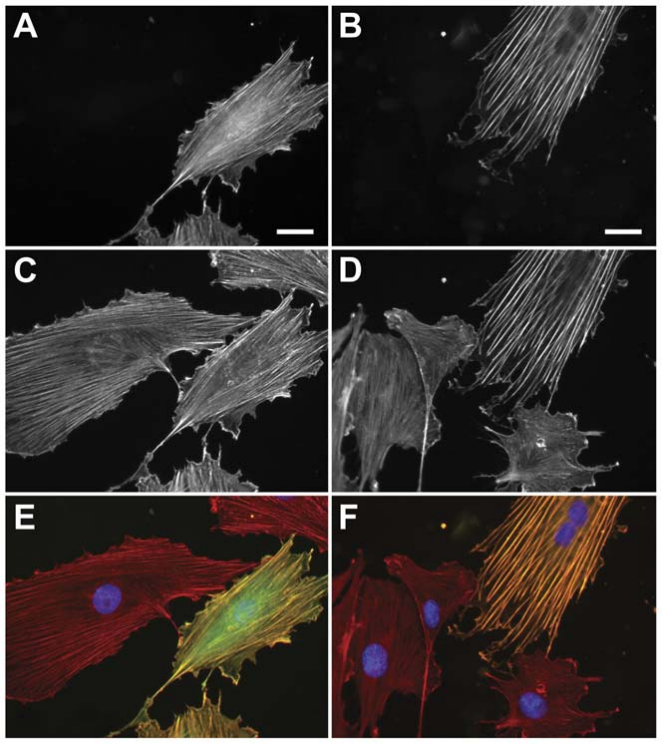
Morphology of pEGFP-Lifeact and pEYFP-actin transfected rat embryonic fibroblast cells (REF52wt). Lifeact (A) and Actin (B) co-localize with Alexa647-phallodin staining (C,D). Phalloidin staining and EGFP-Lifeact expressing cells are corresponding well in their actin morphology. Cells expressing the EYFP-actin fusion protein reveal more pronounced stress fibers compared to the surrounding non-transfected cells. (E,F) Merged images. (Scale bar: 25 µm).

**Figure 2:**
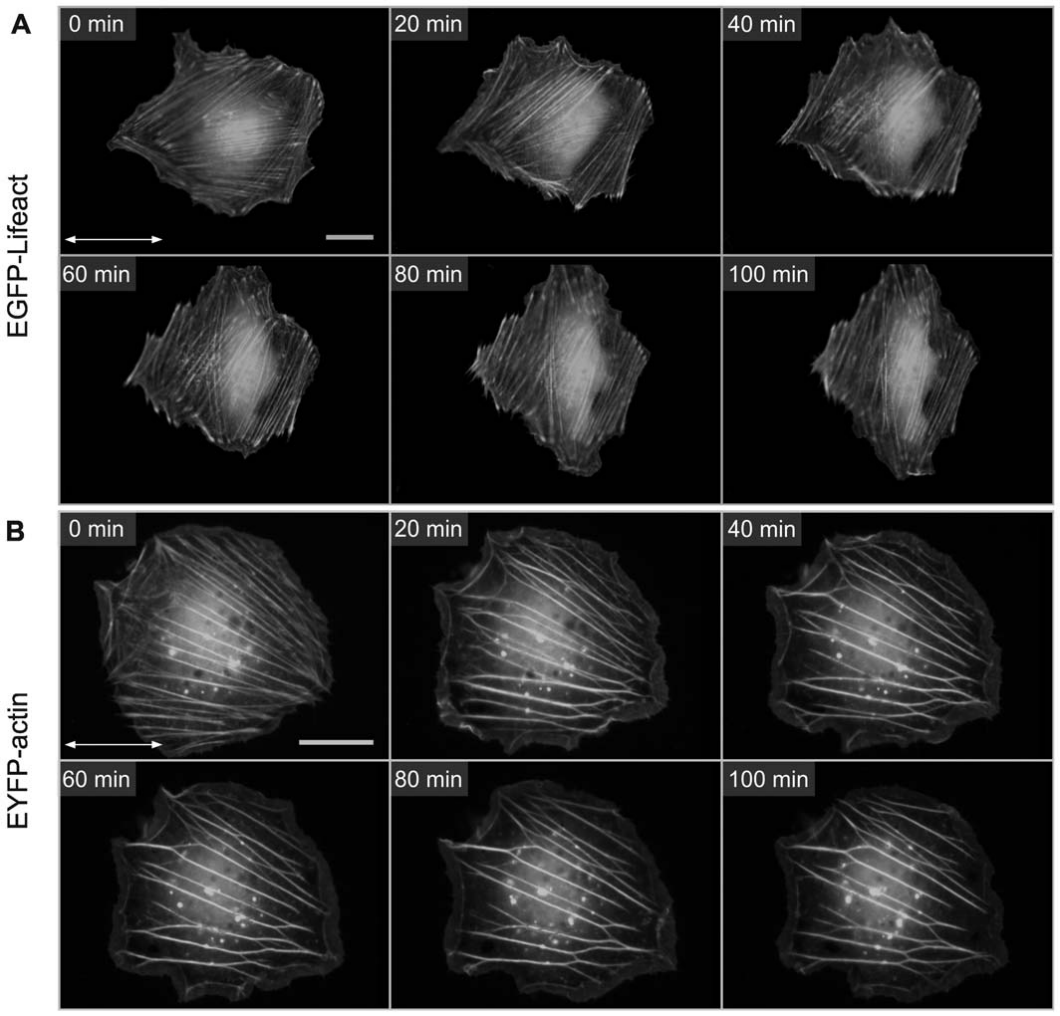
Reorganization of transiently transfected rat embryonic fibroblasts (REF52wt) upon mechanical stimulation. Time series of (A) EGFP-Lifeact and (B) EYFP-actin expressing cells subjected to 4 s^−1^, 8% unaxial cyclic tensile strain. The double arrow indicates the stretch direction (Scale bars: 25 µm). EGFP-Lifeact cells reorganize their actin stress fiber in a perpedicular direction over time. Stress fibers in EYFP-actin cells exhibit a different response by fusing thicker bundles which get partly brigded by other fibers. See supplemntary movies (S1, S2) for further details of the reorganization processes.

Based on our single cell approach in combination with quantitative image analysis we were even able to identify differences between *egfp-lifeact* and *eyfp-actin* expressing cells in static conditions (no stretch). EGFP-Lifeact cells displayed multiple thin stress fibers exhibiting no apparent difference to the morphology of phalloidin-stained microfilaments in non-transfected cells (Fig. 1A, C, E, Fig. 2A). In contrast, actin stress fibers formed pronounced bundles in *eyfp-actin* expressing cells (Fig. 1B, D, F Fig. 2B) indicating an altered phenotype when compared to the thin stress fiber structures in adjacent phalloidin-labeled control cells (Fig. 1B, D, F). Additionally a high degree of alignment and larger inter-fiber-spacing was observable in EYFP-actin cells (Fig. 1B). These findings were substantiated by quantification of the mean inter-fiber-distance in EGFP-Lifeact and EYFP-actin cells. Lifeact-labeled and phalloidin-stained microfilaments possess a significantly smaller average inter-stress-fiber distance (<d_Lifeact_> = 1.6±0.7 µm, <d_Phalloidin_> = 1.2±0.6 µm, SD) than directly labeled, EYFP-actin filaments (<d_Actin_> = 3.2±1.5 µm, SD) (U<0.001, Mann-Whitney test).

**Figure 3:**
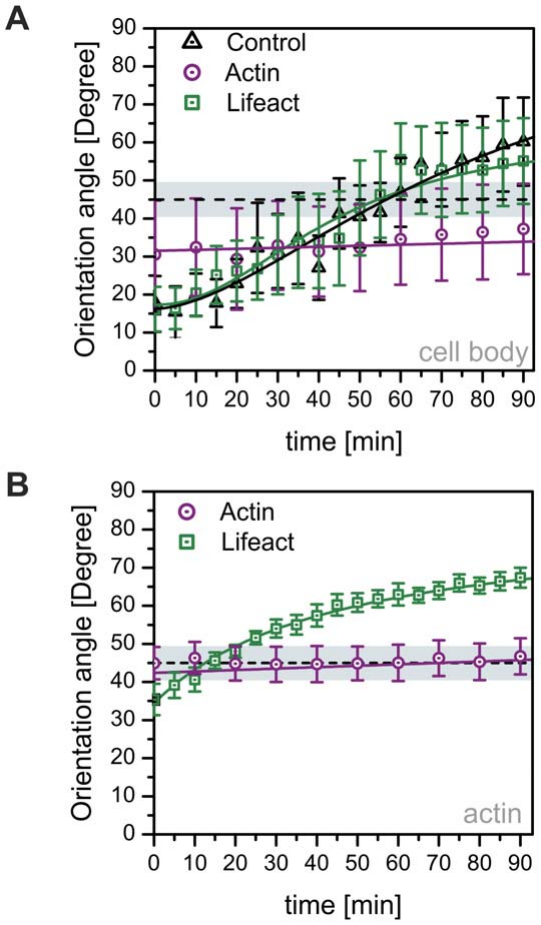
Quantification of cell body and microfilament reorientation kinetics in EGFP-Lifeact and EYFP-actin transfected cells. Average orientation of the (A) cell body and the (B) actin stress fibers with respect to the direction of applied strain over a time period of 90 minutes. Experimental data are represented by the symbols; the solid line indicates a fit of a sigmoidal function to the data. EYFP-actin transfected cells display considerably altered reorientation behavior compared to control and EGFP-Lifeact cells. Dashed line and surrounding gray box indicate regime of indifferent orientation. Data are averages from six cells taken from independent experiments ± s.e.m.

Subjected to periodic tensile strain, EGFP-Lifeact cells showed the characteristic reorientation of the cell body to a mechanically less stressed direction, quasi-perpendicular to the stretching axis (Fig. 2A, Movie S1). This general response is expected considering previous studies [20], [21]. The mean orientation of the cell bodies is altered from an average angle of <ϕ>≈17° to a new direction of approximately 60° with a sigmoidal temporal characteristic within 90 minutes. The initial orientation is set by the deliberate selection of cells having initially a nearly parallel orientation with respect to the stretch direction. Remarkably, the temporal behavior of the Lifeact cells is consistent with the temporal reorientation of non-transfected cells (Fig. 3A). Both, non-transfected and EGFP-Lifeact cells alter their mean cell orientation with a sigmoidal characteristics at nearly the same rate.

Accordingly, actin stress fibers in Lifeact-cells gradually reorganize from an incipient parallel orientation, with respect to the direction of the applied stretch, to a virtually perpendicular orientation (Fig. 2A) by realigning pre-existing fibers. Quantification of the mean stress fiber orientation yields an initial mean orientation of <ϕ>≈35.4°±4.1°. Within 90 minutes the mean stress fiber orientation shifts to <ϕ>≈67.4°±4.0° (Fig. 3B, n = 6 cells) approaching a new steady state. Displaying no initial lag-time, the time course could be fit with a simple exponential function. Closer examination of this stress avoidance response exhibited two major underlying mechanisms – a phenomenological rotation of apparently intact stress fibers away from the major axis of tensile strain and fusion of fibers parallel to the direction of stretch with subsequent alignment along rotating actin filaments (Movie S1).

In contrast, EYFP-actin expressing cells responded neither with a significant reorganization (gray box Fig. 3A, B: regime of indifferent orientation) of the cell body (Fig. 2B, 3A, Movie S2) nor with the expected shift in mean stress fiber orientation (Fig. 3B) in the observed time frame. The stress fibers rather fused to thicker bundles then gradually rotating away from the axis of tensile strain, showing, on average, no pronounced reorganization (Fig. 2B, 3B). After the onset of uniaxial CTS, branching points occurred between adjacent stress fibers leading to fusion and a decrease in the total number of visible microfilaments. Only very few EYFP-actin cells exhibited a small degree of reorganization away from the stretching direction not affecting the average behavior. Due to the experimental outline, stress fibers dynamics of non-transfected cells could not be investigated during mechanical stimulation.

## Discussion

Our results demonstrate that fluorescent actin-fusion proteins are able to alter overall morphology and the dynamic behavior of microfilaments. Significant differences in the stress fiber spacing were visible between *egfp-Lifeact* and *eyfp-actin* expressing cells. Mechanically induced actin stress fiber reorganization exhibits very different kinetics in transiently labeled EGFP-Lifeact and EYFP-actin cells. Only the EGFP-Lifeact cells show a remarkable agreement with the temporal characteristics of reorganization of non-transfected cells (Fig. 3A). Therefore, we present strong evidence that the choice of fluorescent actin marker can fundamentally influence the results of biomechanical studies, particularly when dynamic processes are the focus.

Available studies on time-lapse microscopy investigating the phenomena of stress fiber realignment under uni-axial periodic tensile strain share the common feature of applying either chemically labeled actin or actin fusion proteins in order to track selected fiber populations over time [13], [14], [17], [21]. A holistic comparative approach of analyzing the kinetics of the whole cellular microfilament network and comparing the results with respect to different labeling techniques was not yet applied to biomechanical experiments.

Even though the underlying reason for the different dynamics of the labeled stress fibers is not fully understood we propose that previously reported apparent bundling and shortening [13] of stress fibers exposed to periodic tensile strain might be partly traced back to the effects introduced by the actin fusion proteins. Binding and sliding of non-muscle myosin II within stress fibers might be altered by integration of EYFP-actin chimeras. This interference could induce changes in the stress fiber ultra-structure thereby leading to local shortening effects or enhanced bundling activity. This hypothesis is supported by the findings of Westphal and Aizawa [22], [23]. They suggest that sliding of actin filaments along immobilized heavy meromyosin was reduced in in GFP-actin containing microfilaments. This effect was shown to be dependent on the ratio of labeled actin to unlabeled actin. Lifact in contrast exhibits only low affinity for F-actin, thereby reducing undesired perturbations of protein interactions and functions [10]. This property is likely to be the key for maintaining the dynamical properties necessary for fast structural adaptations. Despite the fact that actin fusion proteins still remain an important tool for investigating the microfilament system, their use in biomechanical studies needs careful consideration. Especially for the investigation of fast force-induced cytoskeleton reorganization, Lifeact might be the better choice.

## Materials and Methods

### Cell culture

REF52wt rat embryonic fibroblasts (kindly provided by the Geiger lab, Weizmann Institute, Israel) were cultured at 37°C, 5% CO2 in Dulbecco's modified eagle medium (DMEM, 4.5 g/L D-glucose) (Invitrogen, Karlsruhe, Germany) supplemented with 10% fetal bovine serum (FBS; Invitrogen). Cells were used before passage 30.

### Plasmids and Transfection

Transfection was performed using pEGFP-Lifeact [10] (kind gift of Michael Sixt, Institute of Science and Technology, Klosterneuburg, Austria) or pEYFP-ß-actin (Clontech, Mountain View, USA) in combination with the Nucleofector Kit R (Lonza, Basel, Switzerland) and the AMAXA Nucleofector system (Lonza). Following transfection, cells were plated on transparent, elastic poly(dimethylsiloxane) (PDMS, Sylgard 184, Dow Corning, Midland, USA) membranes coated with 5 µg/ml bovine fibronectin (Sigma, Steinheim, Germany) and cultured for 16 h at standard conditions (37°C, 5% CO2 ) before experimental use.

### Immunocytochemistry

Transfected and native cells were fixed with 4% paraformaldehyde (Sigma) in PBS and permeabilized with 0.1% Triton-X100/PBS (Sigma). The microfilament network was labeled with Alexa647-phallodin (Invitrogen). Images were acquired with an AxioCam MRm3 CCD-camera on an AxioImager upright microscope equipped with a W-Plan-Apochromat 63x/1.0 objective and an HXP120 illumination system (all Carl Zeiss, Jena, Germany).

### Stretching Experiments

Uniaxial cyclic stretch experiments were performed in phenol red free Leibovitz L-15 media (Invitrogen) supplemented with 5% FBS and 1% penicillin-streptomycin (Invitrogen) at 37°C. A stretching device was customized to fit an upright fixed-stage microscope (AxioExaminer, Carl Zeiss). Uniaxial cyclic stretching was performed according to Jungbauer et al. [19] at 4.0 s^−1^. The stretching amplitude was kept constant at 8% stretch. Imaging was carried out using a W-Plan-Apochromat 40x/1.0 DIC objective (Carl Zeiss) in combination with the Colibri (Carl Zeiss) LED illumination system. Additional magnification was achieved via a manual magnification changer adjacent to the CCD-camera (AxioCam MRm3, Carl Zeiss). Cells with comparable fluorescence intensity were chosen for each experiment. A self-developed software routine was used to synchronize image acquisition with the stretching control. In brief, cyclic stretching was stopped every five and ten minutes respectively, cells were automatically focused and z-stacks were taken in the relaxed state of the substrate.

### Image Processing

Z-stacks were projected via an extended depth of field routine developed for ImageJ [24] by the Biomedical Imaging Group, EPFL, Lausanne [25] and contrast enhanced, setting 0.5% of the overall pixels to saturation. After background removal by means of thresholding and masking, an ellipse was fitted to the contour of the cell, whereby the long axis of the ellipse defined the orientation of the cell dipole with respect to the major axis of deformation. Following this process, actin orientation was extracted by 32×32 pixel sliding square analysis from the segmented and masked images. Fast Fourier Transformation (FFT) was performed in each square following shape analysis of the FFT image by fitting an ellipse to the Fourier spectra and calculating the angle of the major axis. Rotation by 90 degrees yielded the mean orientation of the actin bundles within the field of analysis. The mean angle of actin orientation and the mean standard error was calculated using Matlab (MathWorks, Natick, USA). The mean inter-stress-fiber distance was quantified by means of line plots across five sections within one cell measuring the distance of 13 to 50 fibers for each cell. The results were averaged over six independent experiments.

## Supporting Information

Movie S1 **REF52wt transfected with pEGFP-Lifeact.** Time-lapse images of the fluorescent actin cytoskeleton were taken every 5 minutes over 100 minutes. Cyclic stretching was performed at 4 s^−1^ and 8% uni-axial stretch (application of stretch along the x-axis).
(AVI)

Movie S2 **REF52wt transfected with pEYFP-Actin.** Time-lapse images of the fluorescent actin cytoskeleton were taken every 10 minutes over 100 minutes. Cyclic stretching was performed at 4 s^−1^ and 8% uni-axial stretch (application of stretch along the x-axis).
(AVI)
